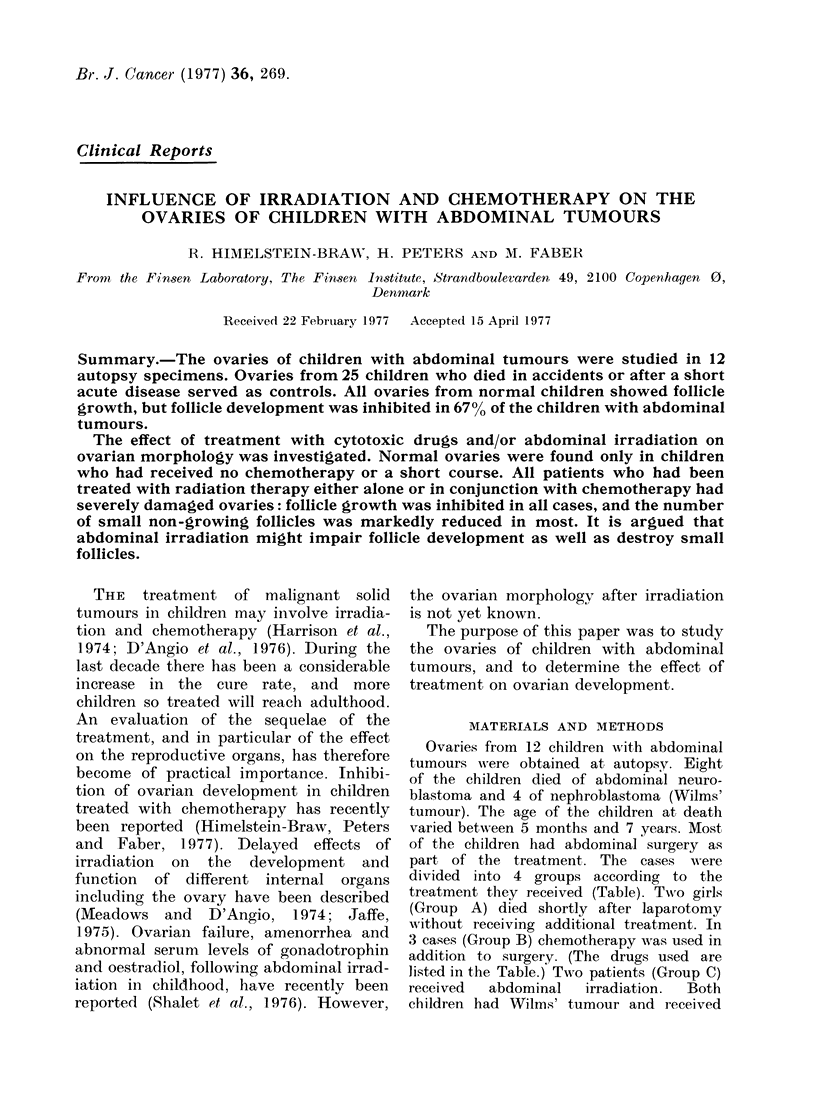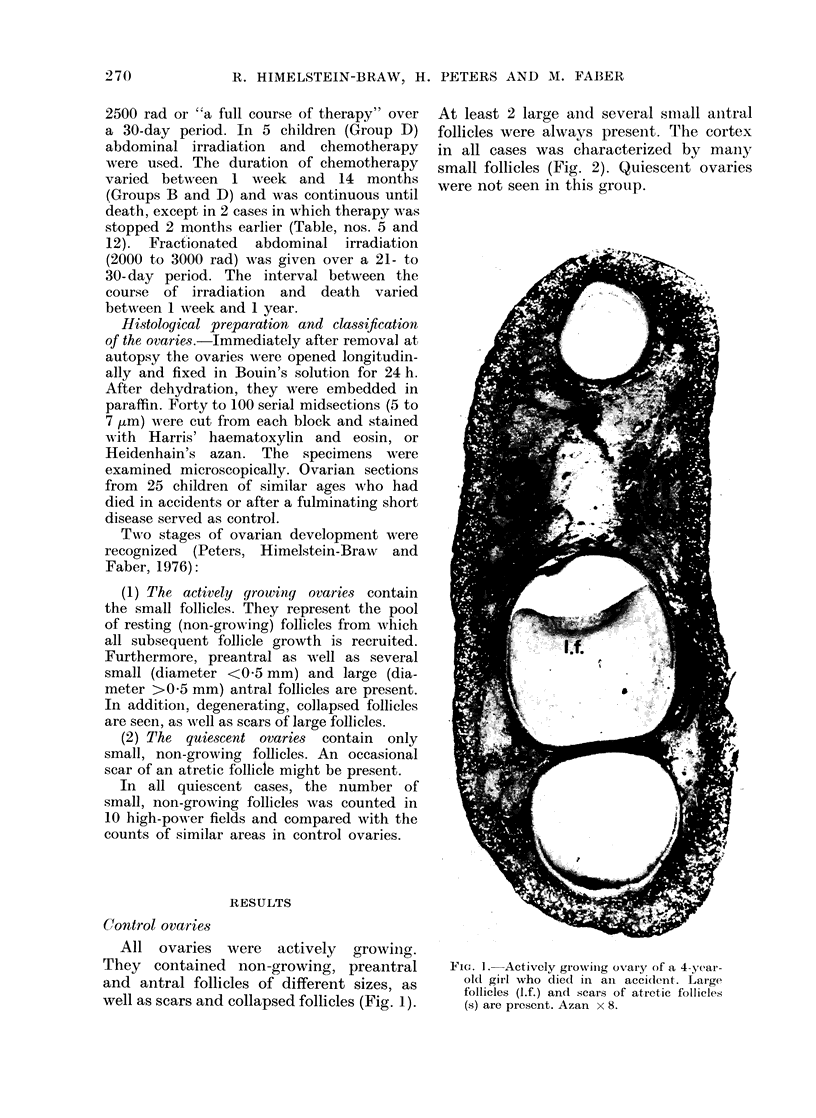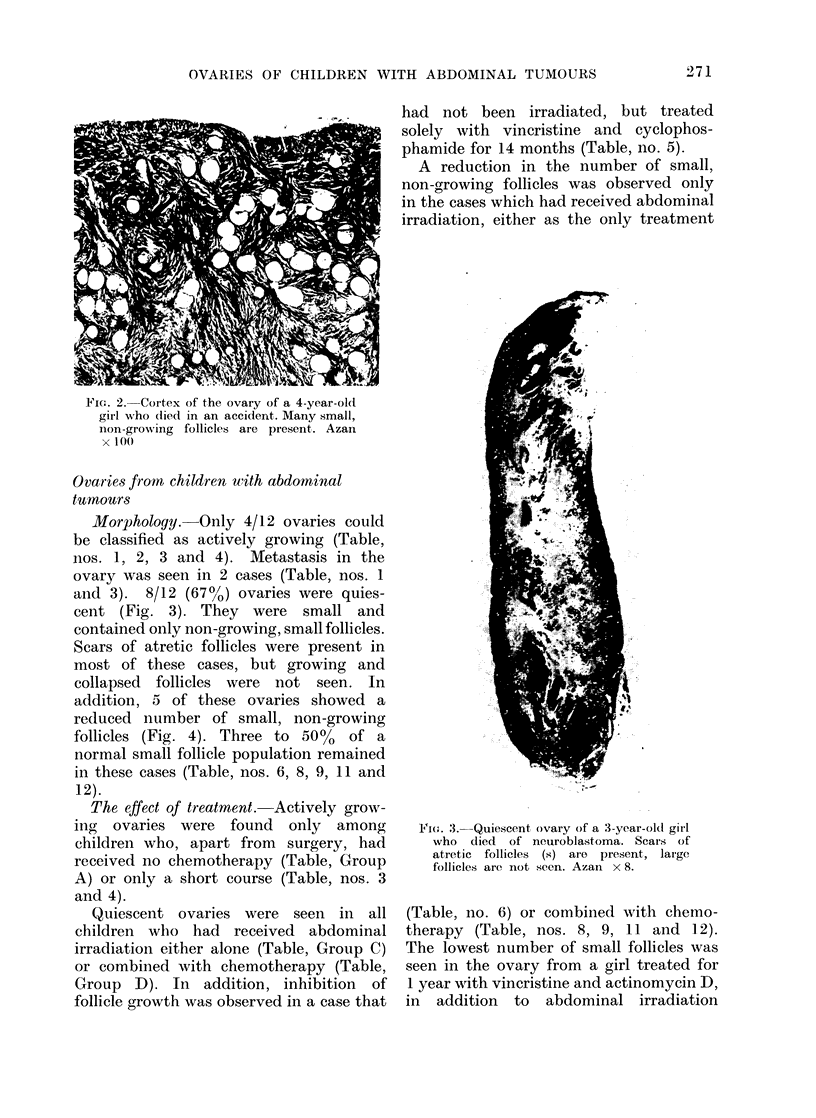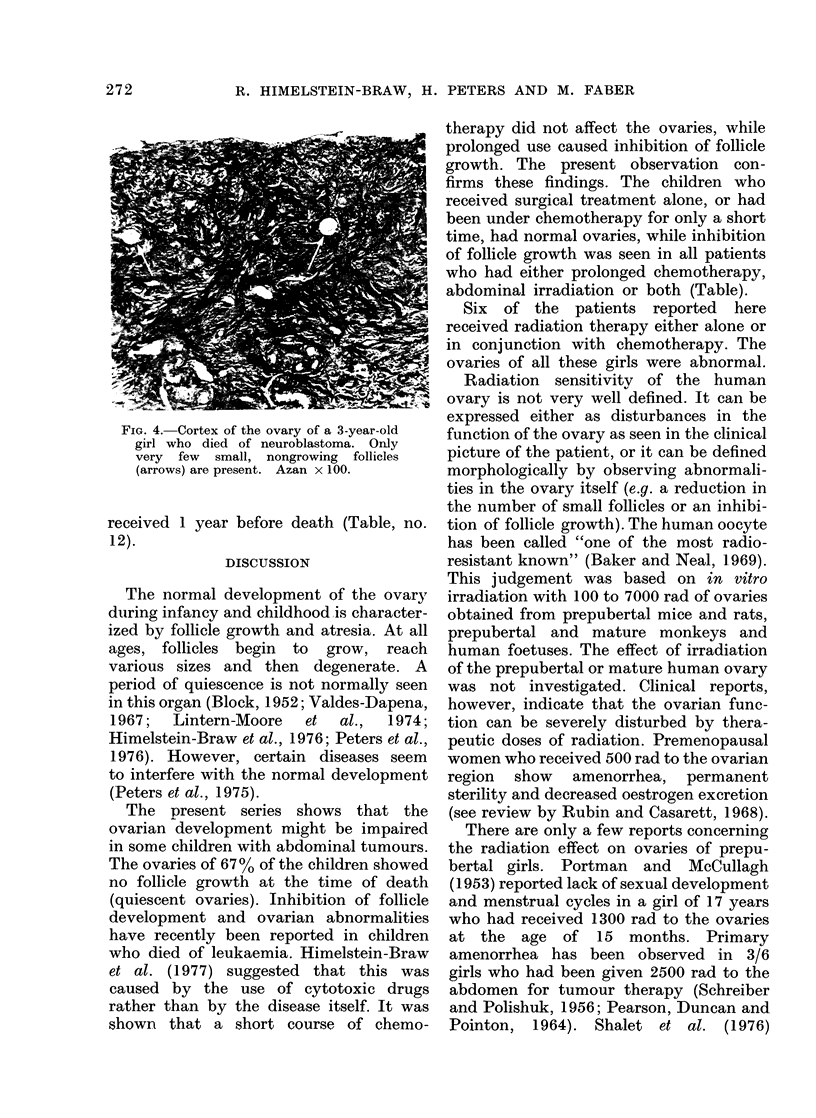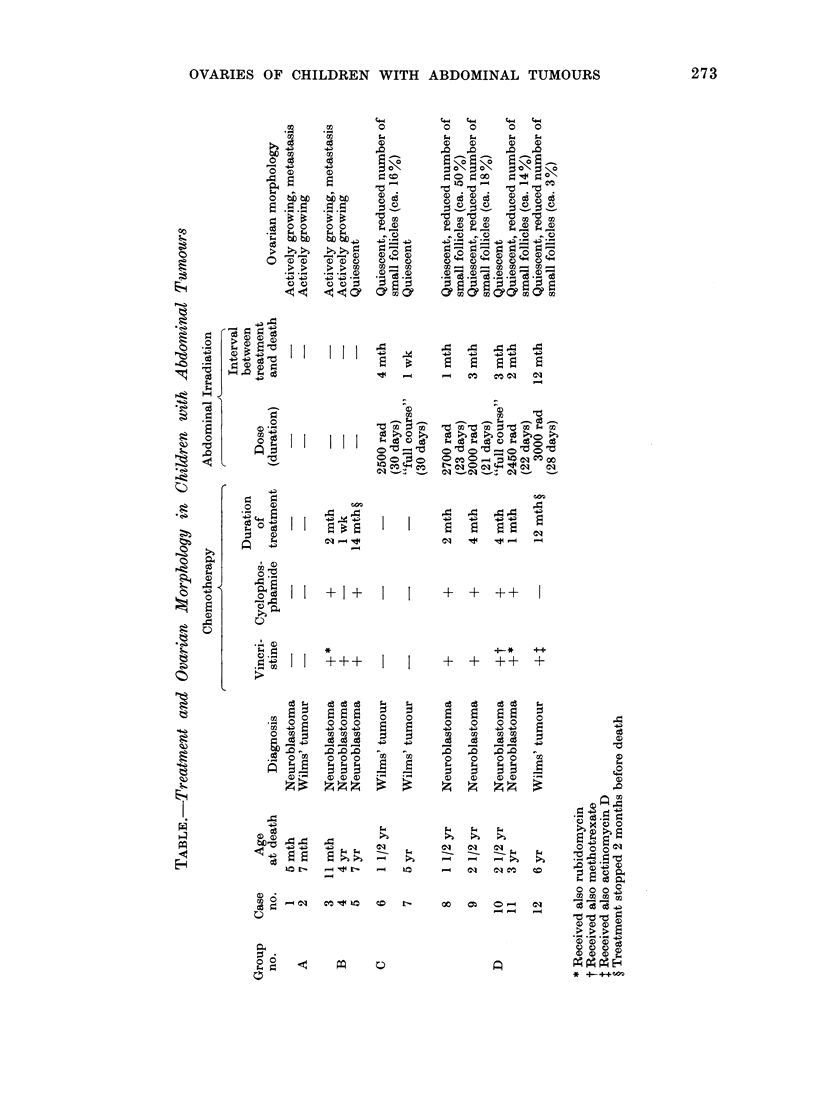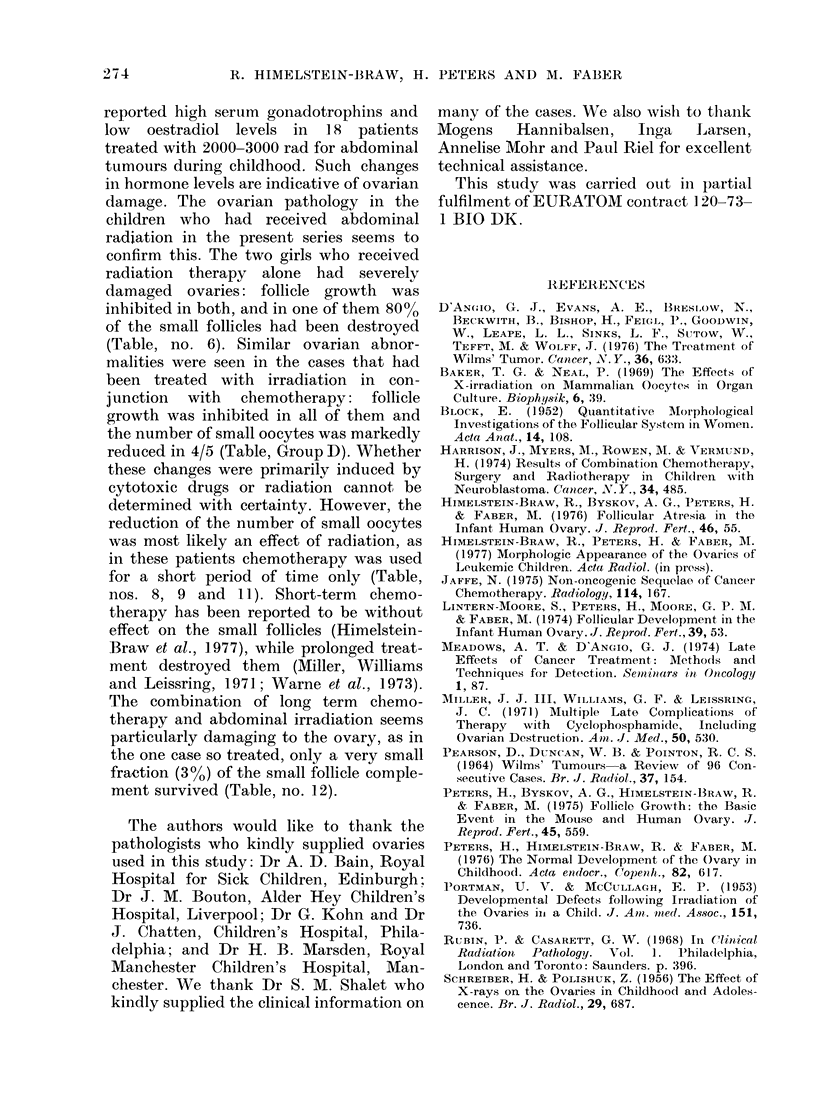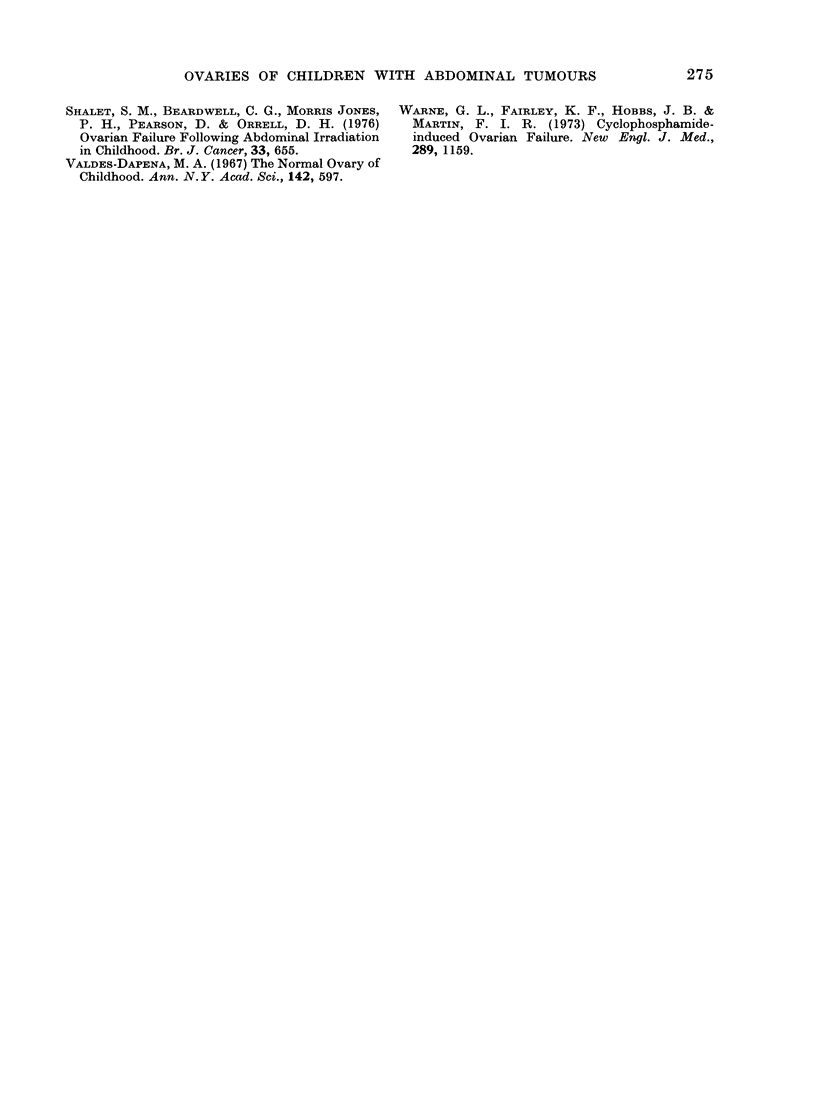# Influence of irradiation and chemotherapy on the ovaries of children with abdominal tumours.

**DOI:** 10.1038/bjc.1977.186

**Published:** 1977-08

**Authors:** R. Himelstein-Braw, H. Peters, M. Faber

## Abstract

**Images:**


*Br. J. Cancer* (1977) **36**, 269.

### *Clinical Reports*

### INFLUENCE OF IRRADIATION AND CHEMOTHERAPY ON THE OVARIES OF CHILDREN WITH ABDOMINAL TUMOURS

R. HIMELSTEIN-BRAW, H. PETERS AND M. FABER

*From the Finsen Laboratory, The Finsen Institute, Strandboulevarden* 49, 2100 *Copenhagen Ø, Denmark*

Received 22 February 1977 Accepted 15 April 1977

**Summary.—The ovaries of children with abdominal tumours were studied in 12 autopsy specimens. Ovaries from 25 children who died in accidents or after a short acute disease served as controls. All ovaries from normal children showed follicle growth, but follicle development was inhibited in 67% of the children with abdominal tumours.**

**The effect of treatment with cytotoxic drugs and/or abdominal irradiation on ovarian morphology was investigated. Normal ovaries were found only in children who had received no chemotherapy or a short course. All patients who had been treated with radiation therapy either alone or in conjunction with chemotherapy had severely damaged ovaries: follicle growth was inhibited in all cases, and the number of small non-growing follicles was markedly reduced in most. It is argued that abdominal irradiation might impair follicle development as well as destroy small follicles.**

THE treatment of malignant solid tumours in children may involve irradiation and chemotherapy (Harrison *et al.*, 1974; D'Angio *et al.*, 1976). During the last decade there has been a considerable increase in the cure rate, and more children so treated will reach adulthood. An evaluation of the sequelae of the treatment, and in particular of the effect on the reproductive organs, has therefore become of practical importance. Inhibition of ovarian development in children treated with chemotherapy has recently been reported (Himelstein-Braw, Peters and Faber, 1977). Delayed effects of irradiation on the development and function of different internal organs including the ovary have been described (Meadows and D'Angio, 1974; Jaffe, 1975). Ovarian failure, amenorrhea and abnormal serum levels of gonadotrophin and oestradiol, following abdominal irradiation in childhood, have recently been reported (Shalet *et al.*, 1976). However, the ovarian morphology after irradiation is not yet known.

The purpose of this paper was to study the ovaries of children with abdominal tumours, and to determine the effect of treatment on ovarian development.

### MATERIALS AND METHODS

Ovaries from 12 children with abdominal tumours were obtained at autopsy. Eight of the children died of abdominal neuroblastoma and 4 of nephroblastoma (Wilms' tumour). The age of the children at death varied between 5 months and 7 years. Most of the children had abdominal surgery as part of the treatment. The cases were divided into 4 groups according to the treatment they received (Table). Two girls (Group A) died shortly after laparotomy without receiving additional treatment. In 3 cases (Group B) chemotherapy was used in addition to surgery. (The drugs used are listed in the Table.) Two patients (Group C) received abdominal irradiation. Both children had Wilms' tumour and received

2500 rad or "a full course of therapy" over a 30-day period. In 5 children (Group D) abdominal irradiation and chemotherapy were used. The duration of chemotherapy varied between 1 week and 14 months (Groups B and D) and was continuous until death, except in 2 cases in which therapy was stopped 2 months earlier (Table, nos. 5 and 12). Fractionated abdominal irradiation (2000 to 3000 rad) was given over a 21- to 30-day period. The interval between the course of irradiation and death varied between 1 week and 1 year.

*Histological preparation and classification of the ovaries.*—Immediately after removal at autopsy the ovaries were opened longitudinally and fixed in Bouin's solution for 24 h. After dehydration, they were embedded in paraffin. Forty to 100 serial midsections (5 to 7 μm) were cut from each block and stained with Harris' haematoxylin and eosin, or Heidenhain's azan. The specimens were examined microscopically. Ovarian sections from 25 children of similar ages who had died in accidents or after a fulminating short disease served as control.

Two stages of ovarian development were recognized (Peters, Himelstein-Braw and Faber, 1976):

(1) *The actively growing ovaries* contain the small follicles. They represent the pool of resting (non-growing) follicles from which all subsequent follicle growth is recruited. Furthermore, preantral as well as several small (diameter <0·5 mm) and large (diameter >0·5 mm) antral follicles are present. In addition, degenerating, collapsed follicles are seen, as well as scars of large follicles.

(2) *The quiescent ovaries* contain only small, non-growing follicles. An occasional scar of an atretic follicle might be present.

In all quiescent cases, the number of small, non-growing follicles was counted in 10 high-power fields and compared with the counts of similar areas in control ovaries.

### RESULTS

### *Control ovaries*

All ovaries were actively growing. They contained non-growing, preantral and antral follicles of different sizes, as well as scars and collapsed follicles (Fig. 1). At least 2 large and several small antral follicles were always present. The cortex in all cases was characterized by many small follicles (Fig. 2). Quiescent ovaries were not seen in this group.

Fig. 1.—Actively growing ovary of a 4-year-old girl who died in an accident. Large follicles (l.f.) and scars of atretic follicles (s) are present. Azan ×8.

FIG. 2.—Cortex of the ovary of a 4-year-old girl who died in an accident. Many small, non-growing follicles are present. Azan ×100

*Ovaries from children with abdominal tumours*

*Morphology.*—Only 4/12 ovaries could be classified as actively growing (Table, nos. 1, 2, 3 and 4). Metastasis in the ovary was seen in 2 cases (Table, nos. 1 and 3). 8/12 (67%) ovaries were quiescent (Fig. 3). They were small and contained only non-growing, small follicles. Scars of atretic follicles were present in most of these cases, but growing and collapsed follicles were not seen. In addition, 5 of these ovaries showed a reduced number of small, non-growing follicles (Fig. 4). Three to 50% of a normal small follicle population remained in these cases (Table, nos. 6, 8, 9, 11 and 12).

*The effect of treatment.*—Actively growing ovaries were found only among children who, apart from surgery, had received no chemotherapy (Table, Group A) or only a short course (Table, nos. 3 and 4).

Quiescent ovaries were seen in all children who had received abdominal irradiation either alone (Table, Group C) or combined with chemotherapy (Table, Group D). In addition, inhibition of follicle growth was observed in a case that had not been irradiated, but treated solely with vincristine and cyclophosphamide for 14 months (Table, no. 5).

A reduction in the number of small, non-growing follicles was observed only in the cases which had received abdominal irradiation, either as the only treatment

FIG. 3.—Quiescent ovary of a 3-year-old girl who died of neuroblastoma. Scars of atretic follicles (s) are present, large follicles are not seen. Azan ×8.

(Table, no. 6) or combined with chemotherapy (Table, nos. 8, 9, 11 and 12). The lowest number of small follicles was seen in the ovary from a girl treated for 1 year with vincristine and actinomycin D, in addition to abdominal irradiation

FIG. 4.—Cortex of the ovary of a 3-year-old girl who died of neuroblastoma. Only very few small, nongrowing follicles (arrows) are present. Azan × 100.

received 1 year before death (Table, no. 12).

DISCUSSION

The normal development of the ovary during infancy and childhood is characterized by follicle growth and atresia. At all ages, follicles begin to grow, reach various sizes and then degenerate. A period of quiescence is not normally seen in this organ (Block, 1952; Valdes-Dapena, 1967; Lintern-Moore *et al.*, 1974; Himelstein-Braw *et al.*, 1976; Peters *et al.*, 1976). However, certain diseases seem to interfere with the normal development (Peters *et al.*, 1975).

The present series shows that the ovarian development might be impaired in some children with abdominal tumours. The ovaries of 67% of the children showed no follicle growth at the time of death (quiescent ovaries). Inhibition of follicle development and ovarian abnormalities have recently been reported in children who died of leukaemia. Himelstein-Braw *et al.* (1977) suggested that this was caused by the use of cytotoxic drugs rather than by the disease itself. It was shown that a short course of chemotherapy did not affect the ovaries, while prolonged use caused inhibition of follicle growth. The present observation confirms these findings. The children who received surgical treatment alone, or had been under chemotherapy for only a short time, had normal ovaries, while inhibition of follicle growth was seen in all patients who had either prolonged chemotherapy, abdominal irradiation or both (Table).

Six of the patients reported here received radiation therapy either alone or in conjunction with chemotherapy. The ovaries of all these girls were abnormal.

Radiation sensitivity of the human ovary is not very well defined. It can be expressed either as disturbances in the function of the ovary as seen in the clinical picture of the patient, or it can be defined morphologically by observing abnormalities in the ovary itself (*e.g.* a reduction in the number of small follicles or an inhibition of follicle growth). The human oocyte has been called "one of the most radio-resistant known" (Baker and Neal, 1969). This judgement was based on *in vitro* irradiation with 100 to 7000 rad of ovaries obtained from prepubertal mice and rats, prepubertal and mature monkeys and human foetuses. The effect of irradiation of the prepubertal or mature human ovary was not investigated. Clinical reports, however, indicate that the ovarian function can be severely disturbed by therapeutic doses of radiation. Premenopausal women who received 500 rad to the ovarian region show amenorrhea, permanent sterility and decreased oestrogen excretion (see review by Rubin and Casarett, 1968).

There are only a few reports concerning the radiation effect on ovaries of prepubertal girls. Portman and McCullagh (1953) reported lack of sexual development and menstrual cycles in a girl of 17 years who had received 1300 rad to the ovaries at the age of 15 months. Primary amenorrhea has been observed in 3/6 girls who had been given 2500 rad to the abdomen for tumour therapy (Schreiber and Polishuk, 1956; Pearson, Duncan and Pointon, 1964). Shalet *et al.* (1976)

TABLE.—*Treatment and Ovarian Morphology in Children with Abdominal Tumours*

| | | | | Chemotherapy | | | Abdominal Irradiation | | |
|---|---|---|---|---|---|---|---|---|---|
| Group no. | Case no. | Age at death | Diagnosis | Vincristine | Cyclophosphamide | Duration of treatment | Dose (duration) | Interval between treatment and death | Ovarian morphology |
| A | 1 | 5 mth | Neuroblastoma | — | — | — | — | — | Actively growing, metastasis |
| | 2 | 7 mth | Wilms' tumour | — | — | — | — | — | Actively growing |
| B | 3 | 11 mth | Neuroblastoma | +* | + | 2 mth | — | — | Actively growing, metastasis |
| | 4 | 4 yr | Neuroblastoma | + | — | 1 wk | — | — | Actively growing |
| | 5 | 7 yr | Neuroblastoma | + | + | 14 mth§ | — | — | Quiescent |
| C | 6 | 1 1/2 yr | Wilms' tumour | — | — | — | 2500 rad (30 days) | 4 mth | Quiescent, reduced number of small follicles (ca. 16%) |
| | 7 | 5 yr | Wilms' tumour | — | — | — | "full course" (30 days) | 1 wk | Quiescent |
| D | 8 | 1 1/2 yr | Neuroblastoma | + | + | 2 mth | 2700 rad (23 days) | 1 mth | Quiescent, reduced number of small follicles (ca. 50%) |
| | 9 | 2 1/2 yr | Neuroblastoma | + | + | 4 mth | 2000 rad (21 days) | 3 mth | Quiescent, reduced number of small follicles (ca. 18%) |
| | 10 | 2 1/2 yr | Neuroblastoma | +† | + | 4 mth | "full course" | 3 mth | Quiescent |
| | 11 | 3 yr | Neuroblastoma | +* | + | 1 mth | 2450 rad (22 days) | 2 mth | Quiescent, reduced number of small follicles (ca. 14%) |
| | 12 | 6 yr | Wilms' tumour | +‡ | — | 12 mth§ | 3000 rad (28 days) | 12 mth | Quiescent, reduced number of small follicles (ca. 3%) |

* Received also rubidomycin

† Received also methotrexate

‡ Received also actinomycin D

§ Treatment stopped 2 months before death reported high serum gonadotrophins and low oestradiol levels in 18 patients treated with 2000–3000 rad for abdominal tumours during childhood. Such changes in hormone levels are indicative of ovarian damage. The ovarian pathology in the children who had received abdominal radiation in the present series seems to confirm this. The two girls who received radiation therapy alone had severely damaged ovaries: follicle growth was inhibited in both, and in one of them 80% of the small follicles had been destroyed (Table, no. 6). Similar ovarian abnormalities were seen in the cases that had been treated with irradiation in conjunction with chemotherapy: follicle growth was inhibited in all of them and the number of small oocytes was markedly reduced in 4/5 (Table, Group D). Whether these changes were primarily induced by cytotoxic drugs or radiation cannot be determined with certainty. However, the reduction of the number of small oocytes was most likely an effect of radiation, as in these patients chemotherapy was used for a short period of time only (Table, nos. 8, 9 and 11). Short-term chemotherapy has been reported to be without effect on the small follicles (Himelstein-Braw *et al.*, 1977), while prolonged treatment destroyed them (Miller, Williams and Leissring, 1971; Warne *et al.*, 1973). The combination of long term chemotherapy and abdominal irradiation seems particularly damaging to the ovary, as in the one case so treated, only a very small fraction (3%) of the small follicle complement survived (Table, no. 12).

The authors would like to thank the pathologists who kindly supplied ovaries used in this study: Dr A. D. Bain, Royal Hospital for Sick Children, Edinburgh; Dr J. M. Bouton, Alder Hey Children's Hospital, Liverpool; Dr G. Kohn and Dr J. Chatten, Children's Hospital, Philadelphia; and Dr H. B. Marsden, Royal Manchester Children's Hospital, Manchester. We thank Dr S. M. Shalet who kindly supplied the clinical information on many of the cases. We also wish to thank Mogens Hannibalsen, Inga Larsen, Annelise Mohr and Paul Riel for excellent technical assistance.

This study was carried out in partial fulfilment of EURATOM contract 120–73–1 BIO DK.

### REFERENCES

D'Angio, G. J., Evans, A. E., Breslow, N., Beckwith, B., Bishop, H., Feigl, P., Goodwin, W., Leape, L. L., Sinks, L. F., Sutow, W., Tefft, M. & Wolff, J. (1976) The Treatment of Wilms' Tumor. *Cancer, N.Y.*, **36**, 633.

Baker, T. G. & Neal, P. (1969) The Effects of X-irradiation on Mammalian Oocytes in Organ Culture. *Biophysik*, **6**, 39.

Block, E. (1952) Quantitative Morphological Investigations of the Follicular System in Women. *Acta Anat.*, **14**, 108.

Harrison, J., Myers, M., Rowen, M. & Vermund, H. (1974) Results of Combination Chemotherapy, Surgery and Radiotherapy in Children with Neuroblastoma. *Cancer, N.Y.*, **34**, 485.

Himelstein-Braw, R., Byskov, A. G., Peters, H. & Faber, M. (1976) Follicular Atresia in the Infant Human Ovary. *J. Reprod. Fert.*, **46**, 55.

Himelstein-Braw, R., Peters, H. & Faber, M. (1977) Morphologic Appearance of the Ovaries of Leukemic Children. *Acta Radiol.* (in press).

Jaffe, N. (1975) Non-oncogenic Sequelae of Cancer Chemotherapy. *Radiology*, **114**, 167.

Lintern-Moore, S., Peters, H., Moore, G. P. M. & Faber, M. (1974) Follicular Development in the Infant Human Ovary. *J. Reprod. Fert.*, **39**, 53.

Meadows, A. T. & D'Angio, G. J. (1974) Late Effects of Cancer Treatment: Methods and Techniques for Detection. *Seminars in Oncology* **1**, 87.

Miller, J. J. III, Williams, G. F. & Leissring, J. C. (1971) Multiple Late Complications of Therapy with Cyclophosphamide, Including Ovarian Destruction. *Am. J. Med.*, **50**, 530.

Pearson, D., Duncan, W. B. & Pointon, R. C. S. (1964) Wilms' Tumours—a Review of 96 Consecutive Cases. *Br. J. Radiol.*, **37**, 154.

Peters, H., Byskov, A. G., Himelstein-Braw, R. & Faber, M. (1975) Follicle Growth: the Basic Event in the Mouse and Human Ovary. *J. Reprod. Fert.*, **45**, 559.

Peters, H., Himelstein-Braw, R. & Faber, M. (1976) The Normal Development of the Ovary in Childhood. *Acta endocr., Copenh.*, **82**, 617.

Portman, U. V. & McCullagh, E. P. (1953) Developmental Defects following Irradiation of the Ovaries in a Child. *J. Am. med. Assoc.*, **151**, 736.

Rubin, P. & Casarett, G. W. (1968) In *Clinical Radiation Pathology*. Vol. 1. Philadelphia, London and Toronto: Saunders. p. 396.

Schreiber, H. & Polishuk, Z. (1956) The Effect of X-rays on the Ovaries in Childhood and Adolescence. *Br. J. Radiol.*, **29**, 687.

SHALET, S. M., BEARDWELL, C. G., MORRIS JONES, P. H., PEARSON, D. & ORRELL, D. H. (1976) Ovarian Failure Following Abdominal Irradiation in Childhood. *Br. J. Cancer*, **33**, 655.

VALDES-DAPENA, M. A. (1967) The Normal Ovary of Childhood. *Ann. N.Y. Acad. Sci.*, **142**, 597.

WARNE, G. L., FAIRLEY, K. F., HOBBS, J. B. & MARTIN, F. I. R. (1973) Cyclophosphamide-induced Ovarian Failure. *New Engl. J. Med.*, **289**, 1159.